# Analysis of web tracking and geolocation of German-language health websites

**DOI:** 10.1371/journal.pone.0323462

**Published:** 2025-05-15

**Authors:** Patrick Siegle, Monika Pobiruchin, Richard Zowalla

**Affiliations:** 1 Faculty of Informatics, Heilbronn University, Heilbronn, Germany; 2 Fraunhofer Institute for Industrial Engineering IAO, Stuttgart, Germany; University of Naples Federico II: Universita degli Studi di Napoli Federico II, ITALY

## Abstract

Nowadays, websites with health-related content are an essential source of information for consumers to find out about their diagnoses, therapies, but also about prevention aspects. The data protection of website visitors is of particular interest here, as the search for health information may allow conclusions to be drawn about their own illnesses or those of their relatives. With a main focus on privacy-relevant aspects, this study examines more than 231,000 German-language health websites from Germany, Austria, and Switzerland. Of all successfully visited websites 56.04% stored at least one cookie. Up to 18.93% of the websites contained cookies that were not technically necessary and were set without explicit consent. The geolocation analysis revealed that among 16.30% of resolved hostnames, at least one IP address was detected that was not from the country suggested by the top-level domain.

## Introduction

Today, governments, cities, municipalities, or institutes publish relevant health information for the public through their own websites [1,2]. Especially during the COVID-19 pandemic important health information, rules and restrictions were quickly disseminated online [2]. Prior to COVID-19, the internet was already being used increasingly for medical and health-related information aimed at everyone [3–6]. Accordingly, in the DACH region (Germany, Austria, and Switzerland) almost 7 out of 10 persons use the internet to obtain information on health topics [7–9].

Health details about an individual are highly intimate and thus particularly worthy of protection – in real life as well as online. In addition to verified accuracy, reliability and comprehensibility, websites with health information or any sources or processors of health information are also expected to respect and protect personal data and privacy to a special extent [5,10–14]. This refers both to technical data such as the IP address, which is considered personal data according to the European General Data Protection Regulation (GDPR), and to potentially actively entered data on the website (e.g., age, gender, known illnesses). In particular, with regard to the sensitivity of the information provided, it is not immediately obvious that websites will attempt to track users across multiple visits, create a unique profile of a user, or improperly store or share data with third parties. In the context of this study, the country associated with an IP address is of particular importance. If the IP address of a web server belongs to a certain country, local data protection laws may apply to the stored and processed data. Ideally, the physical location of the internet host should match the top-level domain (TLD) of a website. A website of the DACH region should comply to GDPR rules and Swiss rules. A mismatch between the DACH region and non-European geolocation could imply an insufficient level of data privacy. The extent to which the web pages employ methods that create a unique user profile or attempt to track the web page visitor across multiple other web pages is also not negligible in terms of data privacy.

### Related work

In the context of health information websites, McCoy et al. conducted a web tracking analysis in 2020 using a tool called webXray [2]. They examined 538 web pages with information on COVID-19 and 89% stored third-party cookies. Two years later, Friedman also used webXray to examine 223 U.S. abortion clinic websites in more detail [15]. They found that third-party data transfers were present on 99.1% of the websites and 69.1% set at least one third-party cookie. Yu et al. focused on 19,483 hospital websites from over 150 different countries with OpenWPM and found trackers on 53.5% of them [16]. In 2023, Friedman et al. looked at a set of 3,747 hospital websites in the U.S. with webXray [17]: They found data transfers to third parties on 98.6% of all websites and 94.3% of them stored at least one third-party cookie.

Conversely, other studies focused on government websites, for example, using either OpenWPM or custom software tools [1,18]. Furthermore, OpenWPM has also been used in several studies to investigate cookie banners and whether they work correctly in different countries [19–21].

Determining the geolocation of internet hosts has been an active area of research for decades. Different approaches have been established, either measuring network latency and thus inferring distances between hosts [22,23] or additionally including network topology [24,25]. Furthermore, alternative proposals were published that combined and improved different geolocation techniques or used further data not directly related to the network [26,27].

### Aims of the study

The aim of this study was three-fold:

1. Assess the prevalence of 3rd party cookies in health-related websites.
2. Assess the prevalence of fingerprinting for web tracking usage in health-related websites.
3. Assess the geolocation of health-related websites and determine possible mis-matches to the website’s domain (TLD).

## Materials and methods

### The German health web

This study utilizes the corpus generated by Zowalla et al. as material for the analyses. The corpus originates from a web-crawl in 2021 and consists of 231,733 hostnames from the TLDs of the DACH countries, namely ‘.de’, ‘.at’, and ‘.ch’. Zowalla et al. designated the considered corpus of German-language web pages with health information as the *German Health Web (GHW)* [28]. They ran the crawler for additional 143 days following their primary publication and published these extended results in 2023 [14]. The collection and analysis methods used in this study complied with the terms and conditions of the data set, which is available on request from the corresponding author.

### Study setting

This study of health-related web pages consisted of three stages:

1. Extract health-related URLs from the GHW’s web graph and check the technical availability of the given web page.
2. Assess the prevalence of 3rd party cookie usage (cf. study aim 1) and fingerprinting (cf. study aim 2) using OpenWPM [29] on the extracted URLs from the GHW.
3. Assess the geolocation (cf. study aim 3) of the extracted URLs from the GHW by using a self implemented framework and relying on well known IP geolocation databases.

### Analysis of web tracking

#### Types of web tracking.

Tracking users in the internet is done either via cookies or fingerprinting methods. The latter attempts to uniquely identify a user (or its browser) without the use of cookies by relying on certain settings, APIs, and device values that vary minimally in browsers.

#### Cookies.

Cookies are small pieces of data that can be set by a special header within an HTTP response of a web server. Each cookie is assigned to a domain, so that browsers send them back only to web servers that are responsible for the associated domain (*first-party cookies*). It is possible that advertising elements embedded on a website may themselves set cookies from another domain. These cookies are called *third-party cookies*.

From a technical perspective, cookies are used in particular for user settings such as the page language or for session handling. However, cookies can also track and identify users. There are services such as *Google Analytics* [30] that can be embedded on a website to analyze users’ behavior. The service *Google AdSense* [31], for example, uses cookies to assess user interests across multiple websites in order to generate personalized advertising [32].

In many countries it is prohibited to set such analysis or tracking cookies without the active consent of a user. For this reason, cookie banners can be found on websites, which ask the user for consent to a wide variety of cookies.

The public Open Cookie Database by Kwakman was used to look up detected cookies [33]. A distinction was made between functional cookies, which cannot be disabled, statistical (i.e., analytics) cookies, and marketing cookies [21]. For additional classification, Disconnect’s Tracking Protection list provided categories such as advertising, analytics, cryptomining, mail, fingerprinting, etc. [34]. Disconnect’s Tracking Protection list has served as the basis for tracking protection in private tabs of the Mozilla Firefox browser since 2015 and for its default tracking protection since 2018 [35].

#### Browser-specific fingerprinting.

OpenWPM is a Python-implemented open source framework for automated web privacy analysis [29]. OpenWPM handles the automated visit of web pages with a browser and is able to record the processes running on them. This includes DNS queries made by the browser, HTTP requests and responses, cookies stored or JavaScript function calls. A customized version of OpenWPM was used for this study. In addition, OpenWPM provides a profile that contains JavaScript interfaces that might be relevant for fingerprinting. It was examined how many properties, such as window.name, window.navigator, etc., potentially relevant to fingerprinting were read from scripts.

#### Canvas fingerprinting.

The same algorithm as proposed by Englehardt and Narayanan was used to detect canvas fingerprinting [36]:

1. the canvas resolution must be at least 16 by 16 pixels,
2. at least two colors or 10 distinct characters must be used to write text on the <canvas > ,
3. the following functions are not supposed to be called: save(), restore(), or addEventListener(), and
4. the drawn result is exported with the method toDataURL() or with a single execution of getImageData(), extracting at least 16 by 16 pixels.

#### Canvas font fingerprinting.

To detect this type of fingerprinting, the approach of Englehardt and Narayanan was followed [36]. The conditions for a script to be associated with canvas font fingerprinting were:

1. the font to draw is changed at least 50 times to unique and valid values,
2. the measureText() method is also invoked at least 50 times with the same text as its argument.

#### Audio fingerprinting.

Due to the slightly changing and partly hardware- and OS-dependent implementations of the individual browsers, there are minimally varying calculation results of audio signals that serve as fingerprint [37]. For the detection of audio fingerprinting, a custom approach was developed in the context of this work. One of the following two conditions must be true:

1. at least 2 AudioNodes are created and linked, the start() method of an AudioNode and the startRendering() method of the OfflineAudioContext are called, and an event listener is added to the OfflineAudioContext waiting for the calculation to complete or
2. at least 3 AudioNodes including an AnalyserNode are created, the destination property of the AudioContext is read at least 1 time, and at least 4 different properties of an AnalyserNode are read.

#### WebRTC fingerprinting.

Another option is to exploit the WebRTC API, which allows real-time communication over the web. WebRTC has a data security vulnerability that can be exploited to determine the local and public IP address of a web page visitor via JavaScript without the user’s active permission and even if they are using a VPN. This information can be abused to create a unique fingerprint or determine a user’s geolocation or network [36,38]. Again, the Englehardt and Narayanan approach was used to detect this type of fingerprinting [36]. For a script to be considered a WebRCT fingerprinting script, the following conditions had to hold:

1. the two methods createDataChannel() and createOffer() of the RTCPeerConnection interface had to be called and
2. an event handler for onicecandidate had to be configured.

#### Automated analysis of web tracking usage.

**Fig 1:**
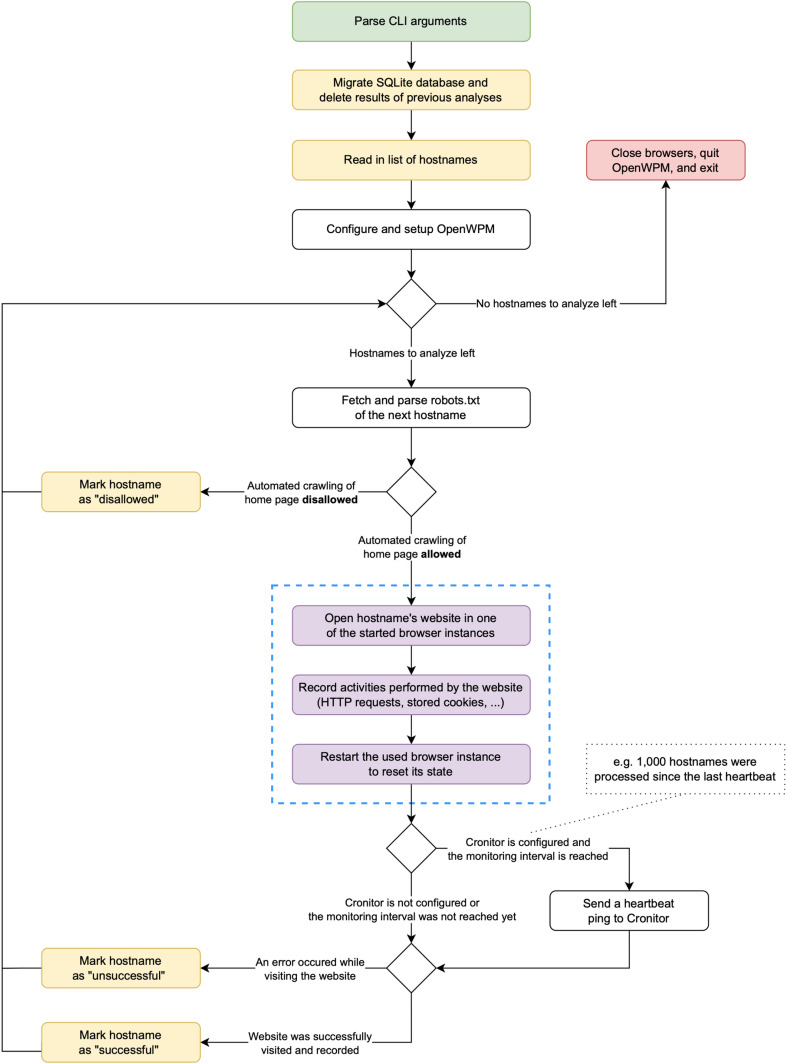
Flowchart of the web tracking analysis tool. Green = starting point; red = endpoint; yellow: I/O task; purple: handled by OpenWPM. The blue dashed box contains the steps that are executed in an independent parallel process managed by OpenWPM.

The procedural flow of the web tracking analysis tool is shown in Fig 1.

The web tracking analysis was performed on a virtual machine at Heilbronn University in Heilbronn, Germany. It had a configuration with 32 virtual CPU of two Intel Xeon Gold 6240Y processors and 48 GB of memory housed in a Cisco UCSB-B200-M5 server. The VM ran Ubuntu Server 22.04.2 LTS as the operating system, not least because OpenWPM officially only supports Ubuntu versions (Englehardt & Narayanan, 2016a). The virtualization software VMware ESXi 7.0.3 was used as hypervisor.

The web tracking analysis tool was run with Python (version 3.10.12) and OpenWPM (version 0.21.1). 32 browser instances were launched, in which the web pages of GHW hostnames were visited in parallel.

The results for each GHW hostname were stored in a SQLLite database thus allowing further analysis of web tracking usage according to the tracking methods described in the section above.

### Analysis of geolocations

Determining the physical location of an internet host based on its IP address, i.e., geolocating its IP address, is a common problem [22,39]. Among measurement- and topology-based approaches, there is also the possibility presented by Mielke and Chen to use geolocation databases [40].Various, usually commercial, providers make such geodatabases publicly available on their websites. A primary distinction is the resolution accuracy, which describes how precisely the location of an IP address can be determined. While the country is the minimal resolution, more precise resolutions can identify states, regions, or even cities. However, the higher the accuracy of a given location, the lower the reliability of this geoinformation [41,42]. In the context of this study, the authors relied on the work by Nishino who provides a GitHub repository that automatically processes the CSV geolocation databases of various providers on a daily basis and makes them available centrally [43].

#### Selection of DNS resolvers.

Popular global as well as German DNS providers were used for the geolocation analysis of GHW hostnames, see Table 1. Accordingly, a total of 7⋅2=14 DNS resolver IP addresses were used to resolve the GHW hostnames to IP addresses in this work. Thus, 14 requests per GHW hostname were made to the DNS resolvers.

**Table 1:** DNS resolver providers for the geolocation analysis including respective IP addresses.

DNS Resolver Provider	1st IP Address	2nd IP Address
Google [44]	8.8.8.8	8.8.4.4
Cloudflare [45]	1.1.1.1	1.0.0.1
OpenDNS [46]	208.67.222.222	208.67.220.220
Quad9 [47]	9.9.9.9	149.112.112.112
CleanBrowsing (Security Filter) [48]	185.228.168.9	185.228.169.9
Freifunk München [49]	5.1.66.255	185.150.99.255
dismail.de [50]	116.203.32.217	159.69.114.157

#### Automated geolocation analysis.

**Fig 2:**
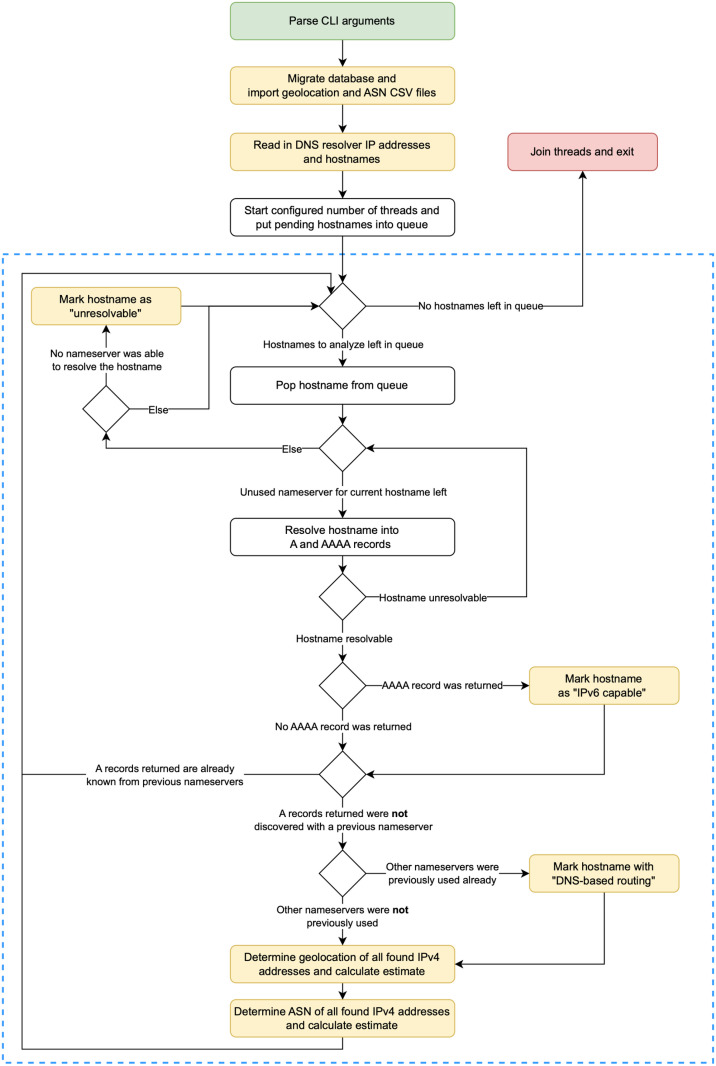
Flowchart of the geolocation analysis tool. Green = starting point; red = endpoint; yellow: I/O task. The blue dashed box contains the steps that can be executed in independent threads whereas the number of threads is configurable.

The analysis of the geolocations of all hostnames of the GHW was performed by a software tool developed for this study written in Python (version 3.10.6). Before and during the analysis, the tool uses PostgreSQL (version 14.7) and is operated entirely through the command line and a configuration file. The tool’s mode of operation is shown in Fig 2.

The geolocation analysis was performed on a virtual machine with Ubuntu Server 22.04.1 LTS as the operating system from the cloud provider *Hetzner* [51]. It was a shared x86-vCPU instance; the machine had 4 virtual cores of a second-generation AMD EPYC CPU, 8 GB of memory, 160 GB of storage and 20 TB of free network traffic included. The city of Falkenstein in Germany was chosen for the server location.

For each IP address, a geolocation estimate was made as to which country it originated from. This estimate was calculated by comparing five different geolocation databases. Each estimate was always given a “certainty value”. The more databases matched in terms of geolocation, the higher the “certainty”. For further analysis of the geolocations of all IP addresses, only those estimates whose certainty was greater than 50% were considered in the following steps.

## Results

The total number of loaded hostnames was 231,733; of these, 327 were ignored because they either contained invalid characters (e.g., commas, wrong UTF-8 encodings), were actually email addresses (e.g., “mail(at)example.com”) or had a double “www” prefix. Hostnames that did not end with a TLD of the DACH region (e.g., ‘.net’) were also ignored.

In total, 231,406 hostnames were analyzed: 191,203 (82.63%) ended with ‘.de’, 21,955 (9.49%) ended with ‘.ch’, and 18,248 (7.89%) ended with ‘.at’. Among these 207,047 could be visited successfully (171,153 for ‘.de’, 19,626 for ‘.at’ and 16,268 for ‘.ch’. Failed website visits could be a result of error during the loading process or server timeouts.

### Analysis of web tracking

#### First-party and third-party cookies.

Among the 171,153 visited German websites, 91,090 (53.22%) stored cookies. Of the 16,268 websites visited in Austria, 14927 (76.06%) stored cookies: 6,989 (42.96%) first-party only, 625 (3.84%) third-party only, and 2391 (14.7%) both types. A total of 19,626 Swiss websites were visited. Of these, 14,927 (76.06%) stored cookies, with 9,289 (47.33%) using only first-party, 612 (3.12%) using only third-party, and 5,026 (25.61%) using both cookie types. Among all successfully visited websites, 116,022 (56.04%) thus set cookies that were not immediately deleted and remained in the browser for at least the duration of a session. These 116,022 web pages have a total number of 492,929 cookies stored. This corresponds to about 4.25 cookies per web page.

Table 2 contains the number of cookies per category determined according to the Open Cookie Database [33].

**Table 2:** Cookie purpose categories by known cookie name according to the “Open Cookie Database” as of August 08, 2023. “Unknown” is referring to cookies with unknown names in the database.

Type	Category	.de	.at	.ch	Total
1^st^ Party	**Persistent**				
	Functional	27,789	3,516	4,592	35,897
	Analytics	47,244	7,045	18,224	72,513
	Marketing	18,998	1,869	5,989	26,856
	Unknown	95,376	11,301	28,240	134,917
	**Session**				
	Functional	21,199	2,529	3,446	27,714
	Analytics	3,757	508	748	5,013
	Marketing	869	100	144	1,113
	Unknown	48,183	4,096	8,006	61,095
3^rd^ Party	**Persistent**				
	Functional	26,568	2,844	6,003	35,415
	Analytics	2,070	192	576	2,838
	Marketing	30,190	2,736	9,147	42,073
	Unknown	21,026	1,604	4,091	26,721
	**Session**				
	Functional	7,287	889	1,250	9,426
	Analytics	358	18	138	514
	Marketing	203	14	56	273
	Unknown	9,603	616	872	11,091

After categorization with Disconnect’s Tracking Protection list [34], it was found that in addition to the above categories, there are two more categories that are not further explained by Disconnect. After manual examination of the hostnames prevailing in this category, the following conclusions about their meaning were drawn by the authors:

- **Content**: Cookies and scripts from domains in this category are used for the technical functioning of a website. They are set, for example, by cloud services on which websites are hosted.
- **Disconnect**: This category includes tracking domains from the three major tech companies *Google*, *Facebook* and *X* (formerly *Twitter*).

Table 3 contains the categorizations of all third-party cookies according to Disconnect’s Tracking Protection list, separated into persistent and session cookies.

**Table 3:** Third-party cookie purpose categories based on Disconnect’s Tracking Protection list. A single cookie can belong to multiple categories.

Category	.de	.at	.ch	Total
**Persistent**				
Advertising	18,678	1,421	5,842	25,941
Analytics	1,368	97	329	1,794
Cryptomining	0	0	0	0
Disconnect	6,369	588	2,243	9,200
Content	19,186	1,988	3,293	24,467
Email	8,483	739	2,659	11,881
Email – Aggressive	310	56	132	498
Fingerprinting – General	7,833	684	2,390	10,907
Fingerprinting – Invasive	4	3	1	8
Session replay	0	0	0	0
Social	3,612	619	2,469	6,700
**Session**				
Advertising	253	17	81	351
Analytics	194	17	45	256
Cryptomining	0	0	0	0
Disconnect	0	0	0	0
Content	4,574	681	1,051	6,306
Email	0	0	0	0
Email – Aggressive	39	0	13	52
Fingerprinting – General	81	8	32	121
Fingerprinting – Invasive	8	2	1	11
Session replay	0	0	0	0
Social	20	2	10	32

#### Browser-specific Fingerprinting.

Table 4 denotes the numbers of websites per DACH country on which at least one script uses a property of the window object presented above. On each website, the script that uses the most unique window properties was considered.

**Table 4:** Numbers of web pages where a script accessed a specific amount of window properties per DACH TLD. Only the script that had the most accesses was counted for each web page.

Use of window Properties	.de	.at	.ch
≥1	91,284	8,202	6,133
≥6	43,301	5,003	9,711
≥10	7,564	851	1,409
≥15	789	66	159
≥20	198	14	23
≥25	320	24	32

From the data in Table 4, it is apparent that 6–9 properties of the window object were used on 58,015 (28.02%) web pages. Between 10 and 14 properties were read on 9,824 (4.74%) web pages. On 1,625 (0.78%) web pages, at least one script used at least 15 window properties. Among them were 376 (0.18%) web pages where at least 25 properties were used, of which 320 (0.19% of all successfully visited German websites) were on German, 24 (0.15%) on Austrian and 32 (0.16%) on Swiss web pages.

Table 5 contains the top 10 URLs of all loaded scripts that performed between 6 and 14 accesses to unique properties of the window object on DACH countries’ websites. In total, these scripts were loaded from 42,213 different unique URLs. Table 6 also shows the top 10 URLs of all loaded scripts which accessed at least 15 unique properties of the window object. Scripts of this type were loaded from 796 unique URLs.

**Table 5:** Top 10 script URLs on GHW web pages of all DACH countries using 6 to 14 different window properties.

Script URL	Using Websites
https://www.google-analytics.com/analytics.js	20,164
https://www.youtube.com/s/player/4cc5d082/www-embed-player.vflset/www-embed-player.js	4,251
https://www.youtube.com/s/player/4cc5d082/player_ias.vflset/en_US/base.js	4,138
https://connect.facebook.net/en_US/fbevents.js	1,903
https://ssl.google-analytics.com/ga.js	1,747
http://www.google-analytics.com/ga.js	1,242
https://www.youtube-nocookie.com/s/player/4cc5d082/www-embed-player.vflset/www-embed-player.js	1,230
https://www.youtube-nocookie.com/s/player/4cc5d082/player_ias.vflset/en_US/base.js	1,213
http://www.google-analytics.com/analytics.js	947
https://bat.bing.com/bat.js	861

**Table 6:** Top 10 script URLs on GHW web pages of all DACH countries using at least 15 different window properties.

Script URL	Using Websites
https://static.adsafeprotected.com/sca.17.6.2.js	167
https://cdn.jsdelivr.net/wp/wp-slimstat/tags/5.0.4/wp-slimstat.min.js	111
https://analytics.webgains.io/pvClk.min.js	101
https://cdn.leadinfo.net/ping.js	99
https://c.paypal.com/da/r/fb.js	61
https://www.clickcease.com/monitor/stat.js	59
https://mc.yandex.ru/metrika/tag.js	53
https://e.video-cdn.net/v2/embed.js	40
https://cdn.jsdelivr.net/wp/wp-slimstat/tags/4.8.8.1/wp-slimstat.min.js	29
https://beagle.prod.tda.link/scripts/newsnet-disco/beagle.min.js	26

#### Canvas Fingerprinting.

At least one script was found on a total of 1,723 (0.83% of all successfully visited web pages) GHW web pages. This included 1,451 (0.85% of a successfully visited web pages from Germany) web pages from Germany, 117 (0.72%) from Austria and 155 (0.79%) from Switzerland.

Table 7 contains the top 10 URLs of all scripts among all successfully visited GHW websites which have performed canvas fingerprinting. In total, such scripts were loaded from 824 different URLs.

**Table 7:** Top 10 script URL on GHW web pages of all DACH countries performing canvas fingerprinting.

Script URL	Using Websites
https://cdn.jsdelivr.net/wp/wp-slimstat/tags/5.0.4/wp-slimstat.min.js	111
https://analytics.webgains.io/pvClk.min.js	101
https://cdn.leadinfo.net/ping.js	99
https://cdnjs.cloudflare.com/ajax/libs/fingerprintjs2/2.1.0/fingerprint2.min.js	82
https://c.paypal.com/da/r/fb.js	61
https://www.clickcease.com/monitor/stat.js	59
https://fast.smarketer.de/api/js/vendors~fp.bundle.js	43
https://e.video-cdn.net/v2/embed.js	40
https://cdn.jsdelivr.net/wp/wp-slimstat/tags/4.8.8.1/wp-slimstat.min.js	29
https://cdn.jsdelivr.net/wp/wp-slimstat/tags/4.9.1.1/wp-slimstat.min.js	20

#### Canvas Font Fingerprinting.

On the GHW web pages, 100 (0.05% of all successfully visited web pages) web pages were found on which canvas font fingerprinting took place. Of these, 83 (0.05% of all successfully visited German web pages) were German, 6 (0.04%) were Austrian, and 11 (0.06%) were Swiss.

Table 8 contains the top 4 URLs of all loaded scripts on the GHW web pages that performed canvas font fingerprinting. All remaining 17 script URLs were only included on one or two web pages, so they were not included in this list.

**Table 8:** Top 4 script URLs on GHW web pages of all DACH countries performing canvas font fingerprinting.

Script URL	Using Websites
https://mc.yandex.ru/metrika/tag.js	53
https://mc.yandex.ru/metrika/watch.js	18
https://cdn.jsdelivr.net/npm/yandex-metrica-watch/tag.js	5
https://mc.yandex.com/metrika/tag.js	4

#### Audio Fingerprinting.

A script performing audio fingerprinting was found on 1,187 (0.57% of all successfully visited web pages) web pages of the GHW. Of these, 1,010 (0.59% of all successfully visited German web pages) were web pages from Germany, 73 (0.45%) from Austria, and 104 (0.53%) from Switzerland.

Table 9 lists the top 10 script URLs from which scripts that performed audio fingerprinting were loaded. In total, such scripts were loaded by 473 unique URLs.

**Table 9:** Top 10 script URLs on GHW web pages of all DACH countries performing audio fingerprinting.

Script URL	Using Websites
https://cdn.jsdelivr.net/wp/wp-slimstat/tags/5.0.4/wp-slimstat.min.js	111
https://analytics.webgains.io/pvClk.min.js	101
https://cdn.leadinfo.net/ping.js	99
https://cdnjs.cloudflare.com/ajax/libs/fingerprintjs2/2.1.0/fingerprint2.min.js	82
https://www.clickcease.com/monitor/stat.js	59
https://fast.smarketer.de/api/js/vendors~fp.bundle.js	43
https://e.video-cdn.net/v2/embed.js	40
https://cdn.jsdelivr.net/wp/wp-slimstat/tags/4.8.8.1/wp-slimstat.min.js	29
https://cdn.jsdelivr.net/wp/wp-slimstat/tags/4.9.1.1/wp-slimstat.min.js	20
https://fast-static.smarketer.de/vendors~fp.bundle.js	18

#### WebRTC Fingerprinting.

Among 136 (0.07% of all successfully visited web pages) web pages of the GHW, the local and public IP addresses of web page visitors might have been determined using the WebRTC API. Of these, 115 (0.07% of all successfully visited German web pages) were German, 9 (0.06%) were Austrian, and 12 (0.06%) were Swiss.

### Analysis of Geolocations

The IP address analysis of the geolocation of all GHW hostnames was started at 9:08 p.m. on May 19, 2023. With a total runtime of 540.2 minutes, the analysis finished at 06:08 a.m. on May 20, 2023. Of all 231,406 hostnames analyzed, 216,533 hostnames (93.57%) could be successfully processed, 14,873 hostnames (6.43%) could not be resolved. The absolute numbers for the distribution of hostnames among the various TLDs as well as their respective status can be found in Table 10.

**Table 10:** Distribution of GHW hostnames by TLD, their corresponding status after the analysis as well as the respective IPv6 support and usage of DNS-based routing.

TLD	Hostnames	Processed	Unresolved	IPv6 Support	DNS Routing
.de	191,203	179,454	11,749	60,256	14,910
.at	18,248	16,888	1,360	2,545	1,399
.ch	21,955	20,191	1,764	4,914	1,996
**Total**	**231,406**	**216,533**	**14,873**	**67,715**	**18,305**

The analysis of DNS-based routing examined whether all the 14 requested DNS resolvers responded with the same IPv4 address for a hostname. If there was more than one IPv4 address returned by a single DNS resolver or any differences among them, the hostname was marked with a flag indicating that DNS-based routing was used. Of all 216,533 hostnames successfully resolved, 18,305 (8.45%) used DNS-based routing. The remaining 198,229 hostnames (91.55%), however, relied on classical routing. With regard to the respective shares per TLD, 9.89% of all hostnames in Switzerland used DNS-based routing, 8.31% in Germany and 8.28% in Austria.

#### Geolocation of IP Addresses.

In total, 277,409 IP addresses were discovered for the 216,533 successfully resolved hostnames. The average “geolocation certainty” among all IP addresses was 89.59%. Of these, 227,782 (82.11%) IP addresses belonged to hostnames with a ‘.de’ ending, 22,015 (7.94%) to ‘.at’-suffixed hostnames and 27,612 (9.95%) to hostnames ending with ‘.ch’. For 1,371 IP addresses, no country of origin could be determined because there was no relative majority among the database votes. Among all IP addresses there were 277,239 (99.94%) valid and 170 (0.06%) invalid IP addresses. Invalid addresses were those listed in RFC6890 as “IPv4 special-purpose addresses”. Table 11 contains the top 15 countries with the respective number of IP addresses localized there.

**Table 11:** Top 15 countries among all discovered IP addresses.

Country	# IP Addresses	%	95% CI
Germany	180,639	67.59	[67.42,67.76]
United States	43,239	16.18	[16.04,16.32]
Switzerland	15,063	5.64	[5.55,5.73]
Ireland	9,769	3.66	[3.59,3.73]
Austria	9,506	3.56	[3.49,3.63]
Canada	2,422	0.91	[0.87,0.95]
Denmark	1,854	0.69	[0.66,0.72]
Netherlands	1,427	0.53	[0.50,0.56]
France	1,128	0.42	[0.40,0.44]
Finland	562	0.21	[0.19,0.23]
United Kingdom	410	0.15	[0.14,0.16]
Sweden	212	0.08	[0.07,0.09]
Czechia	179	0.07	[0.06,0.08]
Australia	150	0.06	[0.05,0.07]
Belgium	138	0.05	[0.04,0.06]

#### Match Between Actual and Suggested IP Address Geolocation.

**Fig 3:**
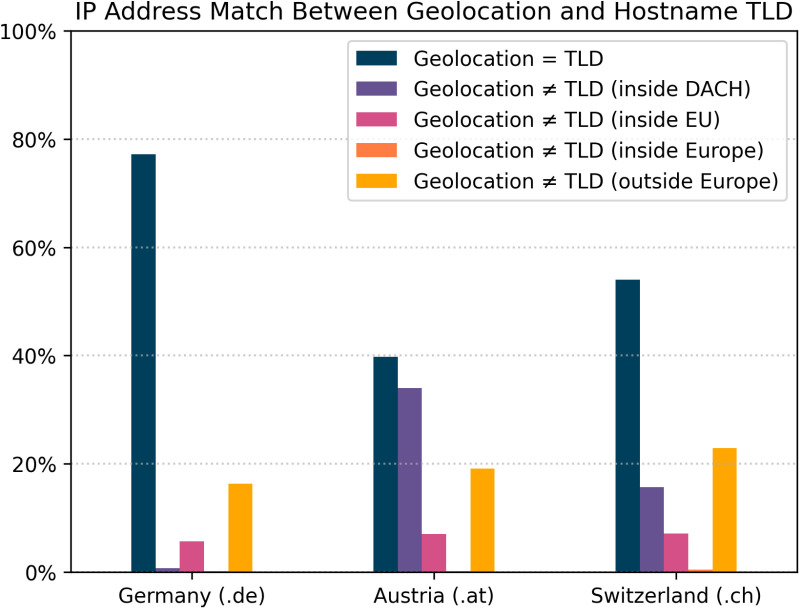
Match between suggested geolocation by TLD and geolocation yielded during the analysis. “Europe” refers to the countries geographically located in Europe, which are, however, not part of EU.

Figure 3 shows the relative proportions of IP addresses whose geolocation matches or does not match the suggested geolocation of the hostname TLD. The latter distinguishes “how much” the geolocation determined in the analysis differs from the suggested geolocation:

- The geolocation does match the geolocation suggested by the TLD (blue).
- The geolocation does not match the geolocation suggested by the TLD of the hostname, but the IP address still belongs to the DACH region (purple),
- the geolocation of the IP address is not in the DACH region, but still in the EU (pink),
- the geolocation of the IP address is no longer in a EU member state but geographically in Europe (orange), or
- the geolocation of the IP address is outside the geographical Europe (yellow).

For hostnames with the German TLD ‘.de’, 169,666 (77.18%) associated IP addresses actually originated from Germany as well. Another 1,644 (0.75%) IP addresses were still located in the DACH region. 12,387 (5.63%) IP addresses originated from the EU, 325 (0.15%) from geographic Europe and 35,819 (16.29%) originated outside Europe.

Of the IP addresses belonging to hostnames with the TLD ‘.at’, 8,346 (39.75%) were actually from Austria. 7,140 (34.01%) were located in the DACH region. The EU region included 1,465 (6.98%) IP addresses, while 35 (0.17%) others belonged to geographic Europe only. Finally, 4,008 (19.09%) IP addresses were located outside Europe.

For Swiss hostnames with the TLD ‘.ch’, 14,270 (53.98%) IP addresses were also located there. 4,142 (15.67%) other IP addresses were still located in the DACH region, 1,872 (7.08%) in the EU, and 107 (0.4%) within geographic Europe. In contrast, 6,047 (22.87%) IP addresses were outside of Europe.

IP addresses that originated from countries that are known for low “level of internet freedom” including violation of user rights (according to [52]) could be traced to Russia with 14, Singapore with 9, and Turkey with 8 IP addresses.

## Discussion

### Principal findings

#### Analysis of Web tracking usage.

Examination of the first- and third-party cookies stored on the GHW web pages revealed, that 36.28% of all web pages set only first-party cookies, 4.14% set only third-party cookies, and 15.61% set both types of cookies. Thus, a total of 56.04% of the GHW websites stored at least one cookie. The results showed that based on the cookie’s name, first- and/or third-party cookies were identified as potential tracking cookies and thus not technically necessary on 18.93% of the GHW websites. The same was true for 4.66% of the web pages based on a tracker list of the company “Disconnect” or 10.69% based on three different hostname blocklists.

Finally, it was investigated how many websites also used techniques to track website visitors beyond the use of cookies. On 32.77% of the web pages, scripts were found that read at least six properties of the window object and thus at least very likely collected data about a user to use it for analysis purposes. On another 0.83%, canvas fingerprinting was detected, on 0.78% more than 15 window properties were read, and on 0.57% audio fingerprinting took place. In addition, 0.06% of the web pages performed WebRTC and 0.05% canvas font fingerprinting. In total, 33.56% of all successfully visited web pages were affected by some kind of fingerprinting or at least by the reading of certain properties for analysis purposes.

#### Geolocation analysis.

The same valid hostnames as used for the web tracking analysis were used for geolocation analysis.

A total of 277,239 valid IP addresses could be found, for which the country of origin was finally determined. The top 5 countries from which most IP addresses originated were Germany (67.59%), the United States (16.18%), Switzerland (5.64%), Ireland (3.66%), and Austria (3.56%).

It could be shown that at least one IP address of 11.39% of the ‘.de’ hostnames could not be traced back to Germany. The same was true for 52.17% of the ‘.at’ and 29.94% of the ‘.ch’ hostnames or 16.30% of all GHW hostnames that could be successfully resolved. Thus, it became clear that the TLD does not necessarily correspond to the location of the responsible server. Moreover, 19,932 (6.93%) IP addresses in the GHW originated from outside Europe.

#### Analysis of geolocations.

The geolocation analysis only took place based on DNS responses. This ignored both whether the website itself was still available at all and whether the content still matched a valid website with German-language health information. The assumption was that a correct DNS response meant that the hostname was still active and the website was still providing relevant content. In a future work, it would also be possible to ensure that a web page with relevant content is still accessible under a GHW hostname beyond the DNS response. These results could also be used to reassess the current state of the GHW determined by [14,28]. Manual examination of some suspected cases showed that this assumption could be valid for a certain part of the GHW. It was also found that some domains are now offered for sale and corresponding hosting companies provide advertisements for purchase in the form of a web page. Not looking at the web page itself also means that only the IP address of the first touch point of a client was examined for its geolocation. Further HTTP requests, which are automatically performed by a web browser when visiting a website to load embedded content such as images, stylesheets or scripts of an HTML document, were thus neglected. However, this content often originates from external, third-party sources under a different domain, which may cause a client to connect to a server in a non-German-speaking region, even though the web server of the originally visited website is located in the DACH region. During the web tracking analysis with OpenWPM, all HTTP requests as well as the respective responses of the DNS servers to the hostnames contained therein were stored in the form of IP addresses. A future work could perform a closer analysis of these hostnames and IP addresses based on the software tool developed in this work to determine the geolocation of web servers delivering third-party content.

The fact that only the IP address returned by a DNS response was used to determine their geolocation lead to further limitations. It was not distinguished for an IP address whether it already points to the web server that also delivers the page content or merely a proxy or, for example, a load balancer of a content delivery network (CDN). During the geolocation analysis, it was only checked if DNS-based routing takes place or is suspected when different DNS returned different IP addresses. In many cases, however, it is not possible to determine from the IP address or the behavior of the DNS to which type of host it points. It is therefore possible that the IP address of a DNS response does not have to point directly to an interface of a web server from which the web page content is ultimately delivered. Nevertheless, the particular geolocation of these IP addresses means that a client establishes at least a network connection to that country, where potentially different privacy policies apply and where at least the IP address of that client is stored. For this reason, the results presented are relevant even for IP addresses that do not directly belong to a web server. It should also be noted that both when first connecting to a web server via a forwarding response and when visiting a web page itself through special HTML tags or JavaScript, there may be a redirect to another hostname located in a different country [53]. These redirections were also neglected in the context of the geolocation analysis against the background that clients nevertheless at least establish a connection to the IP address of a hostname returned by its DNS. In this case, the IP address could be stored, although forwarding occurs after that. A more targeted analysis of the collected IP addresses in the future could attempt to determine the type of host and in which country the web content is initially hosted before it is distributed in a global CDN, for example. It would also be conceivable to include redirects by analyzing responses given from a host behind an IP address for redirection HTTP status codes and HTML-based or JavaScript-based redirection.

Finally, it should be noted that the geolocation analysis was performed throughout from a virtual server located in Germany. Thus, due to the geographic location, DNS-based routing in particular may result in responses and thus IP addresses that would be different from another location. However, since the question revolves around German-language websites and it can be assumed that these are visited particularly in German-speaking countries, the location of the analysis server in Germany is chosen sensibly.

### Comparison with related work

OpenWPM was first introduced by Englehardt and Narayanan as part of a web tracking analysis of the top one million websites, according to the Alexa toolbar, in 2016. During the study, 917,261 of the top one million websites were successfully visited, with more than 81,000 individual third parties identified. Within the GHW, only 5,113 (−75,887) different third parties were detected. Furthermore, canvas fingerprinting was found on 1.57% and canvas font fingerprinting on 0.35% websites. This compared to 0.83% (−0.74%) and 0.05% (−0.3%) of GHW web pages, respectively. WebRTC fingerprinting was found on 0.08% of the top one million websites, while it occurred on 0.06% (−0.02) of the GHW websites. Fingerprinting using the Web Audio API was used on 0.06% of the top one million websites and on 0.57% (+0.51%) in the GHW.

Overall, all other fingerprinting methods studied were found to be used less frequently in GHW than among the top one million Alexa websites. The exception was audio fingerprinting, which was used on 0.51% more websites in the GHW. The top 10 script URLs that performed audio fingerprinting were often loaded by analytic tools that tried to tell website visitors apart even without cookies to count unique page visits. Chalise et al. showed in the past that entropy could be increased by 9.6% by using the Web Audio API in addition to canvas fingerprinting [54]. Thus, it stands to reason that modern fingerprinting scripts would also make increased use of the Web Audio API to compute more unique fingerprints. For the remaining fingerprinting methods, which were less frequently found in the GHW, the introduction of the GDPR in 2018, as well as additional enactments of national privacy laws, have made the use of fingerprinting more strictly regulated. This is also consistent with the findings of Urban et al., which in an analysis of 2,659,873 URLs found a decrease in ID sharing due to the GDPR, showing that it had an impact on websites of operators located in the EU [55]. In addition, Englehardt and Narayanan examined the top one million websites (about 770,000 more than in the GHW) of all users of the Alexa toolbar worldwide, thus allowing websites to originate from various countries in which there were few or no privacy policies regarding tracking and fingerprinting.

Some of the studies presented considered the presence of cookie banners and, occasionally, their correct functioning with the help of OpenWPM [19–21]. In doing so, Kampanos and Shahandashti found that 61% of the 14,650 Greek websites studied and 70% of the 17,737 UK websites stored third-party cookies. Sheil and Malone showed that 57.14% of 3,735 visited Irish websites set third-party cookies. In the GHW, this was the case for only 19.75% of websites. This reduced number of stored third-party cookies might be also again due to the GDPR and national privacy policies regarding cookies in the DACH region. However, at the time of the [19] and [20] studies, the GDPR already applied in the EU member states of Greece and Ireland, so their results are surprising.

Among 30,520 domains studied, Rizzo et al. found at least one form of fingerprinting in 2.76% of the cases [56]. Cheng were able to detect canvas fingerprinting on 18.61% (+17.78 more than in GHW), canvas font fingerprinting on 4.97% (+4.92), audio fingerprinting on 4.12% (+3.55%), and WebRTC fingerprinting on 4.28% (+4.22%) of the Alexa top 10,000 websites visited [57].

Samarasinghe et al. focused on 150,244 websites from 206 different countries, each run by governments [18]. During their investigation with OpenWPM, they found known third-party tracking cookies on 13.7% of the web pages. In the GHW, depending on how the type of cookie was determined, 8.15% (−5.55%) were identified to have potential third-party tracking cookies according to the name database, 4.66% (−9.04) according to Disconnect, and 10.69% (−3.01) according to all hostname block lists. In addition, Samarasinghe et al. identified known trackers on 29.9% of the examined websites [18]. A study of Gotze et al. showed that up to 90% of all websites provided by governments of a G20 country stored third-party cookies without the consent of the website visitor [1]. This is up to 70.25% more than in the GHW.

Similar to the web tracking analysis of the GHW, researchers have also previously examined smaller corpora of health information websites with webXray, as an alternative to OpenWPM, or other custom tools. To the authors’ knowledge, a large-scale study of health-related websites such as the one in this manuscript has never been conducted before using OpenWPM or any other tools. Using webXray, McCoy et al. found third-party cookies on 89% of all visited mainly US-based web pages that provided COVID-19 information [2], 69.25% more than in the GHW. Gotze et al. also examined web pages about COVID-19 and detected third-party cookies on 62% of these [1], 42.25% more than in the GHW. In a study of Friedman et al., third-party cookies were detected on 69.1% of the 223 websites of “National Abortion Federation member facilities” [15] examined in the U.S.. That’s 49.35% websites that stored third-party cookies more than in the GHW. A year later, Friedman et al. examined 3747 “US nonfederal acute care hospital websites.” [17], of which 94.3% stored third-party cookies and thus 74.55% more than in the GHW.

In addition to studies that used tools other than OpenWPM to check the prevalence of tracking on health-related web pages, Yu et al. used OpenWPM to examine a total of 19.483 hospital websites in over 150 countries for deployed tracking scripts and cookies [16]. They detected one of these trackers on 53.5% of the websites.

Compared with all the studies presented that examined health-related websites, fewer trackers and especially fewer third-party cookies were found on average in the GHW. This result shows that privacy and data protection had a higher priority on the GHW websites than on other health information websites studied in the past. The results of all the previously discussed comparative studies that focused on other types of websites also support this observation with regard to tracking such as fingerprinting, with a few exceptions. This is not least due to the legal framework that applied in each case in the DACH region and thus also to the GHW. These include the EU-wide GDPR, the Swiss DSG or revDSG, and other national guidelines, which prohibit the storage of certain cookies without explicit consent. The legal impact on the prevalence of third-party cookies in the GHW was again confirmed by the fact that the studies of other health websites took place in other countries such as the U.S.. Nevertheless, the results for the GHW were surprising as health websites catering to the German-speaking world should be subject to strict privacy policies in most cases. In addition, the privacy of visitors to health-related websites is to be considered particularly worthy of protection in general, since the topics are often personal, e.g., revealing a person’s illness or psychological concerns. Nevertheless, numerous cookies could be identified for tracking purposes, on almost half of all GHW websites at least one HTTP request would have been blocked, and up to one third of all websites also deployed fingerprinting techniques.

### Limitations

#### Uneven representation of hostnames.

The majority of GHW hostnames, 82.63%, ended with ‘.de’; in comparison, 9.49% ended with ‘.ch’ and 7.89% with ‘.at’. The consequence of this imbalance is that the corpus of ‘.de’ hostnames also contains more websites that are run by, e.g., private medical practices or regional pharmacies. Websites of this type have a limited, local audience and are therefore not technically designed for millions of visitors. The same applies to private blogs and websites of individuals or associations.

#### Analysis of web tracking usage.

OpenWPM currently only supports research with an automated Mozilla Firefox instance. As of May 2023, Firefox had a 2.77% market share, while browsers like Google Chrome and Apple Safari collectively accounted for over 80% [58]. Although many web standards are implemented equally by the various developers of the browsers, there are differences that, among other things, have an impact on user privacy [59]. A future work could extend OpenWPM to support more browsers while investigating the differences in the GHW regarding web tracking depending on the browser. Since OpenWPM is based on Selenium’s API, it should be possible to integrate browsers supported by Selenium with acceptable effort.

Before a website was crawled and tested during the web tracking analysis, it was first checked whether automated crawling is allowed at all according to its robots.txt. The analysis tools were implemented using the Python programming language, which also provides the module urllib.robotsparser [60]. This allows robots.txt files to be parsed according to the official, historical syntax of 1994 [61]. More advanced specifications are not covered.

Another limitation is that no consideration was given to the presented content of a website during the web tracking analysis. The GHW was crawled by Zowalla et al. between 2020 and 2021, while the analysis presented here took place in 2023. It is thus possible that website content has changed in the meantime, no longer exists, certain hostnames are no longer accessible, or have a different owner. However, it was assumed that all GHW web pages are still valid and continue to provide health information. The geolocation analysis already suggested that some web pages are no longer available in the form they were during the GHW crawl. Random manual inspection of some of these sites confirmed that affected hostnames have since been parked and/or are being offered for sale. These websites were nevertheless visited by OpenWPM and the results subsequently evaluated.

OpenWPM did not interact with the visited web pages during the web tracking analysis. The respective home page was opened only once and it was waited until all HTTP requests were completed and scripts were executed before the next web page was visited. This procedure was primarily chosen to check if the websites store cookies, load other trackers, or calculate fingerprints without the active consent of a website visitor. Due to the GDPR as well as national regulations, it is necessary to either display a cookie banner where users can only consent to certain types of cookies or, in the case of Switzerland, at least give a hint that cookies are used and how they can be disabled. However, these cookie banners were not interacted with at any time by OpenWPM and therefore no consent was given. Conversely, this approach has three disadvantages. First, it is not possible to determine on which web pages a cookie banner was present and whether they function correctly for certain types of use. As a result, this study does not distinguish between tracking with and without explicit user consent. Consequently, tracking cookies set after user consent were not analysed, leaving a gap in the understanding of post-consent tracking behaviour. In addition, it was not feasible to distinguish between purely informational pages, where users do not make any additional inputs except clicks on links, and interactive web pages, e.g., with login options, other forms, or a forum. On some web pages, it might be possible for users to provide health information in a form (e.g., age, weight, medical history, etc). By their nature, these data would be particularly worthy of protection because they are health-related. However, such website features were not investigated and could be considered in a future work, which could then also address privacy from this perspective. Nevertheless, all websites with health information, regardless of the features they offer, are expected to handle user data responsibly and in particular not to share it with third parties without being asked [10–13]. The importance of privacy plays a role on all websites and especially when users do not disclose any additional information of their own they do not expect that technical data is nevertheless processed in the background to investigate their browsing behavior or to track them across multiple websites.

#### Quality of health information.

This study primarily examines the technical aspects of health information websites, focusing on practices such as the use of tracking cookies and geolocation. However, it does not address the aspect of health information quality, nor does it distinguish between different types of health-related data presented on websites, such as general health information versus patient-specific data. Consequently, it does not assess the reliability, accuracy or credibility of the content presented on these websites, nor does it explore how these factors may relate to privacy concerns. For example, a person seeking information about a stigmatized condition may have heightened concerns about tracking behavior or geolocation.

Given the nature of health data, particularly sensitive patient information, more robust privacy and security measures may be required compared to other types of health-related websites. However, as this aspect was beyond the scope of this study, no conclusions can be drawn. This provides an opportunity for future research into privacy concerns and the quality of health information.

## Conclusions and further research

In this study, geolocations and web tracking usage of websites within the GHW were analyzed. The web tracking analysis revealed that many websites of the GHW used cookies and tracking scripts without user consent, violating data protection laws. The geolocation analysis demonstrated that while most IP addresses could be traced accurately, a notable portion of websites of the GHW were hosted outside their TLD regions, implying potential data protection risks. However, compared with all the studies presented that examined health-related websites, fewer trackers and especially fewer third-party cookies were found on average in the GHW. This result shows that privacy and data protection had a higher priority on the GHW websites than on other health information websites studied in the past. Nevertheless, these findings highlight discrepancies between assumed and actual data protection standards on GHWs, indicating that website visitors cannot reliably infer data protection compliance based on TLDs alone.

Future research can explore several avenues to improve privacy on health-related websites. These include extending geolocation analysis to determine the origin of website content elements and the associated data transfer risks. In addition, legal impact studies are essential to assess compliance with new data protection regulations, such as the Swiss revDSG and the EU-US data protection framework, and to identify persistent legal gaps. In addition, the feasibility of distinguishing between websites that provide only health-related information and those requiring user registration or additional data entry should be explored. Such a distinction would allow for a more nuanced analysis of privacy practices, particularly in relation to sensitive health data and could provide valuable insights.

Moreover, evaluating the effectiveness of privacy-enhancing tools such as VPNs, tracking blockers and privacy-compliant browsers can provide insights into mitigating tracking and privacy issues. Moreover, extending analysis tools to different regions and website types can facilitate global comparisons of privacy practices and regulatory impacts. Furthermore, examining the role of auditing bodies in enforcing privacy laws and exploring strategies for stronger enforcement could ensure higher privacy standards on health-related websites.
